# Correlation between Levels of 2, 5-Hexanedione and Pyrrole Adducts in Tissues of Rats Exposure to *n*-Hexane for 5-Days

**DOI:** 10.1371/journal.pone.0076011

**Published:** 2013-09-30

**Authors:** Hongyin Yin, Ying Guo, Tao Zeng, Xiulan Zhao, Keqin Xie

**Affiliations:** 1 Institute of Toxicology, School of Public Health, Shandong University, Jinan, Shandong Province, China; 2 Jinan Municipal Center for Disease Control & Prevention, Jinan, Shandong Province, China; University of Colorado, Denver, United States of America

## Abstract

**Background:**

The formation of pyrrole adducts might be responsible for peripheral nerve injury caused by *n*-hexane. The internal dose of pyrrole adducts would supply more information for the neurotoxicity of *n*-hexane. The current study was designed to investigate the tissue distributions of 2, 5-hexanedione (2,5-HD) and pyrrole adducts in rats exposed to *n*-hexane, and analyze the correlation between pyrrole adducts and 2,5-HD in tissues.

**Methods:**

Male Wistar rats were given daily dose of 500,1000, 2000, 4000 mg/kg bw *n*-hexane by gavage for 5 days. The rats were sacrificed 24 hours after the last administration. The levels of 2, 5-hexanedione and pyrrole adducts in tissues were measured by gas chromatography and Ehrlich’s reagent, respectively. The correlations between 2, 5-hexanedione and pyrrole adducts were analyzed by linear regression

**Results:**

Dose-dependent effects were observed between the dosage of *n*-hexane and 2, 5-hexanedione, and pyrrole adducts in tissues. The highest level of 2, 5-hexanedione was found in urine and the lowest in sciatic nerve, while the highest level of pyrrole adducts was seen in liver and the lowest in serum. There were significant correlations among the free 2, 5-hexanedione, total 2, 5-hexanedione and pyrrole adducts within the same tissues. Pyrrole adducts in serum showed the most significant correlation with free 2, 5-hexanedione or pyrrole adducts in tissues.

**Conclusion:**

The findings suggested that pyrrole adducts in serum might be a better indicator for the internal dose of free 2, 5-hexanedione and pyrrole adducts in tissues.

## Introduction

*n*-Hexane is a solvent that has many uses in the chemical and food industries. *n*-Hexane exposure could induce peripheral neuropathy characterized by neuromuscular paralysation and sensory alterations [1]. *n*-Hexane is principally metabolized in the liver [2]. It is initially metabolized to either 1-hexanol or 3-hexanol in a detoxification pathway or 2-hexanol in a bio-activation pathway, and then the 2-hexanol is converted to 2-hexanone and 2,5-hexanediol, both of which are further metabolized to 5-hydroxy-2-hexanone, 2, 5-hexanedione (2,5-HD) and 4,5-dihydroxy-2-hexanone. 2, 5-HD is believed to be the major toxic metabolite produced following acid hydrolysis of urine samples prior to analysis by gas chromatography in humans or experimental animals [3,4]. 2,5-HD could react with the lysine ε-amino group and form pyrrole adducts [5-10]. Pyrrole adducts formed, would undergo oxidation to electrophiles and react with protein nucleophiles, resulting in the covalent cross-linking of derivatized proteins to form higher molecular weight protein aggregates [9,11-14]. This step was a critical step for the induction of neurotoxicity by *n*-hexane [7,8,15-17].

In the liver, *n*-hexane is metabolized to 2, 5-HD which is then distributed in the blood to various organs, including the liver, kidney and brain. The internal doses of *n*-hexane and 2,5-HD have been studied in previous study. The highest concentrations of *n*-hexane and 2,5-HD were observed in sciatic nerve and kidney, respectively [18-20]. Due to the critical role of pyrrole adducts in the neurotoxicity of *n*-hexane, the internal dose of pyrrole adducts might provide more information for the neurotoxicity research of *n*-hexane. However, no attempts have been made to clarify the internal dose of pyrrole adducts. In *in vitro* studies, it had been documented that the formation of pyrrole adducts was related to the levels of 2, 5-HD [7,8], while the correlation between 2, 5-HD and pyrrole adducts *in vivo* was still unknown.

Levels of 2,5-HD were divided into free 2,5-HD and total 2,5-HD. Free 2,5-HD refers to the 2,5-HD detected in samples without acid hydrolysis, while the 2,5-HD obtained from acid hydrolysis is called total 2,5-HD. Total 2,5-HD contains the free 2,5-HD (real 2,5-HD) and 2,5-HD produced from 4,5-dihydroxy-2-hexanone, a product of the detoxification of *n*-hexane [21,22]. It had been demonstrated that there were significant correlations between free and total 2,5-HD in urine and serum, and free 2,5-HD was approximately 4.6-47.4% of total 2,5-HD in urine and 50% in serum [23].

Although the internal doses of 2,5-HD and pyrrole adducts might be more representative for the toxic effect of *n*-hexane, the detection of internal doses 2,5-HD and pyrrole adducts of the exposed workers was unable to be carried out. In view of the convenience of sample collection, serum or urine was the most suitable media for detection. However, it was still unknown whether the 2,5-HD and pyrrole adducts in serum or urine could represent those in other tissues.

The current study was designed to investigate the tissue distributions of 2,5-HD and pyrrole adducts in rats, and analyze the correlation between pyrrole adducts and 2,5-HD in tissues.

## Materials and Methods

### Materials

2,5-dimethylpyrrole, 2,5-hexaneketone, trypsin, guanidine hydrochloride, 4-dimethylaminobenzaldehyde and boron fluoride (14% solution) were purchased from Sigma-Aldrich, Inc. (St. Louis, MO, USA). Ethanol, cyclohexanone, dichloromethane, hydrochloric acid were purchased from Hongyan chemical reagent factory (Tianjin, China). All chemicals were of the highest grade commercially available.

### Animals Experiments

#### Ethics Statement

The experiments were conducted in accordance with the NIH Guide for Care and Use of Laboratory Animals and the principles in the “Use of Animals in Toxicology”, and were approved by the Ethics Committee for Animal Experiments of Shandong University Institute of Preventive Medicine (Permit Number: 20130601).

#### Animals

Adult male Wistar rats (Experimental Animal Center of Shandong University), weighing 280-300 g, were used in this experiment. Animals had free access to tap water and standard rat chow. Animal housing and care followed currently accepted standards of the NIH Guide for Care and Use of Laboratory Animals and the principles in the “Use of Animals in Toxicology”. The animal room temperature was maintained at 20 ± 2 °C, with a relative humidity control of 50 ± 10%, and 12 hours light/dark cycle.

#### Animals and Treatments

After 7 days for acclimatization, the animals were randomly divided into five groups (n = 8). Experimental group rats were treated with *n*-hexane by gavage at a dosage of 500, 1000, 2000, 4000 mg/kg/day for 5 days. The control group rats received an equivalent volume of 0.9% saline. Rats were sacrificed under anesthesia with a proper amount of diethyl ether at 24 hours after the last administration of *n*-hexane. Blood and urine samples were collected from abdominal aorta and bladder, respectively. The liver, kidney, spinal cord, brain, and sciatic nerve were quickly removed, snap frozen in liquid nitrogen, and then stored at −80°C until analysis.

### Analytical Methods

#### Free 2,5-HD Detection

A 50 µl of 0.5 mg/ml of cyclohexanone (internal standard) and 0.2 g of NaCl were added to 100 µl urine or serum sample. The 2,5-HD was extracted with 1.0 ml of dichloromethane, followed by rotating of the samples and centrifugation at 3500 rpm for 10 min. The solvent extracted was evaporated to about 0.1 ml with a nitrogen flow and then injected into a gas chromatography system [24]. Tissues were homogenized in physiological saline at 0.5g wet weight/ml, and the homogenates were used for the detection of free 2,5-HD in accordance with the serum or urine [25].

The GC used was a Shimadzu GC-2010 (Kyoto, Japan) equipped with a flame ionization detector (FID) and an HP-5 column (30 m × 0.32 mm × 0.25 µm, Agilent Technologies). The injector temperature was set at 180 °C and the detector temperature at 260 °C. The oven was set at 80 °C and the injection volume was 2 µl. The carrier gas was nitrogen at a flow rate of 1.5 ml/min and the split ratio was set at 3:1.

#### Total 2, 5-HD Detection

The urine, serum and tissue homogenate were hydrolyzed with HCl (pH0.1) at 100°C for 30 min, and the 2, 5-HD formed was detected following the above procedure. Values were expressed as nanomoles of 2,5-HD per milliliter or gram of tissue [6].

#### Pyrrole Adducts Detection

The pyrrole adducts were measured spectrophotometrically after reaction of 0.1 ml of urine or serum with 0.1 ml guanidine hydrochloride (70%) and 0.1 ml of Ehrlich’s reagent (3% 4-dimethylaminobenzaldheyde in 40 ml of methanolic 14% boron trifluoride and 60 ml of ethanol) [26]. Absorption values were measured at 526 nm, using automatic microplate reader (Infinite® 200 PRO, TECAN Inc. Switzer). The calculations were based on standard curve prepared with different concentrations of 2,5-dimethylpyrrole [25].

Liver, kidney, spinal cord and brain samples were homogenized in physiological saline at 0.5g wet weight/ml. A 0.5ml of 0.7g/ml of aqueous guanidine hydrochloride was added to 0.1ml tissue homogenate. After digestion at room temperature for 30 min, the pyrrole adducts was determined by the above procedure [25].

Sciatic nerves were initially broken into powder with pestle in liquid nitrogen [27], and then homogenized in physiological saline at a concentration of 0.5g wet weight/ml, followed by adding 0.3ml of 2.5% trypsin. After digestion at 56 °C for 1 hour, the pyrrole adducts was determined by the above procedure.

Values were expressed as nanomoles of 2,5-dimethylpyrrole per milliliter or or gram of tissue.

### Statistical Analysis

All data were expressed as mean ± S.D. SPSS13.0 statistical software was used for statistical analysis. All data were analyzed using one-way analysis of variance (ANOVA). The correlations were analyzed by linear regression. The differences were considered significant at *P* < 0.05.

## Results

### Tissue Distributions of Free 2, 5-HD, Total 2, 5-HD and Pyrrole Adducts

**Figure 1:**
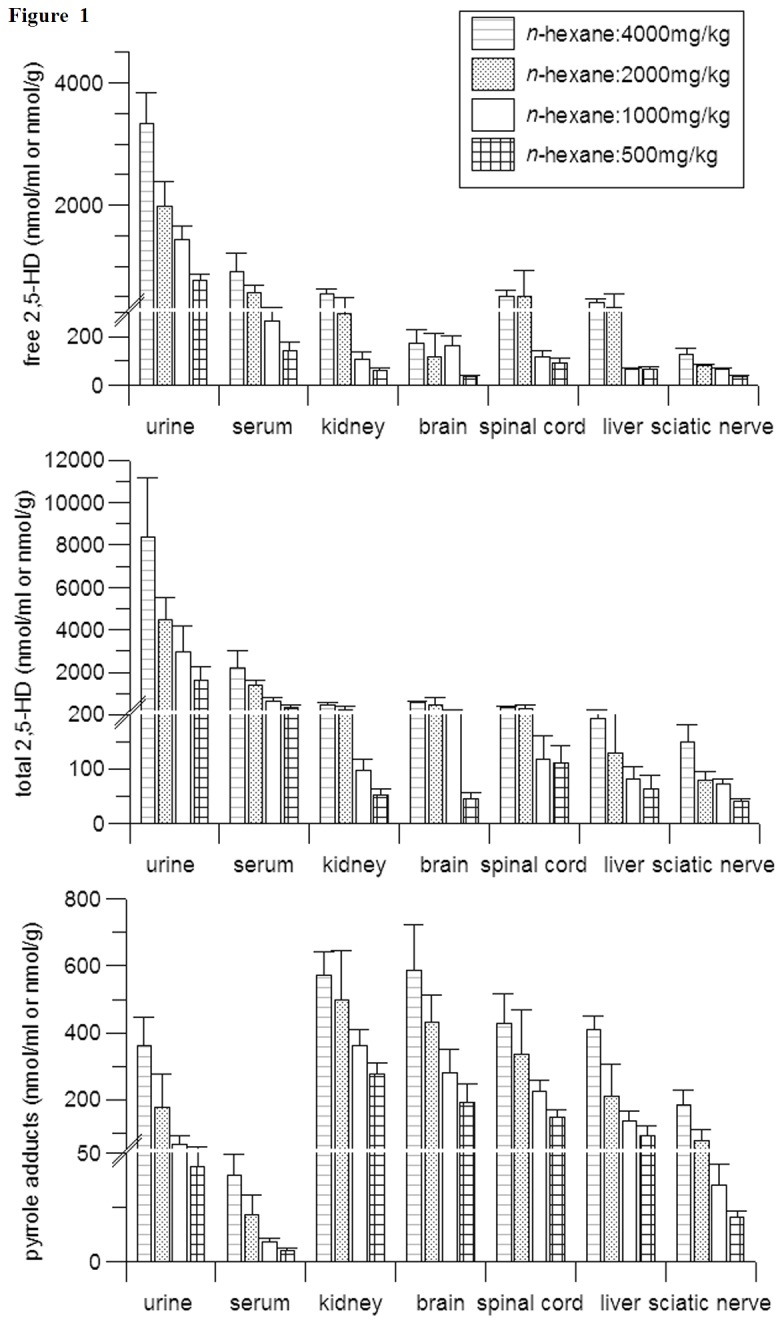
Tissue distributions of free 2,5-HD, total 2,5-HD and pyrrole adducts at 24 hours after the last administration of rats which were given daily dose of 500, 1000, 2000 or 4000 mg/kg *n*-hexane for 5 days (n=8). Free 2, 5-HD and total 2, 5-HD were not detected in tissues of control group rats, the detection limit was 1.0 nmol/ml or nmol/g. Pyrrole adducts in serum and urine were 1.23 ± 0.26, 0.83 ± 0.13 nmol/ml, respectively. Pyrrole adducts were not detected in kidney, liver, spinal cord, brain, sciatic nerve, the detection limit was 0.20 nmol/g.

**Figure 2:**
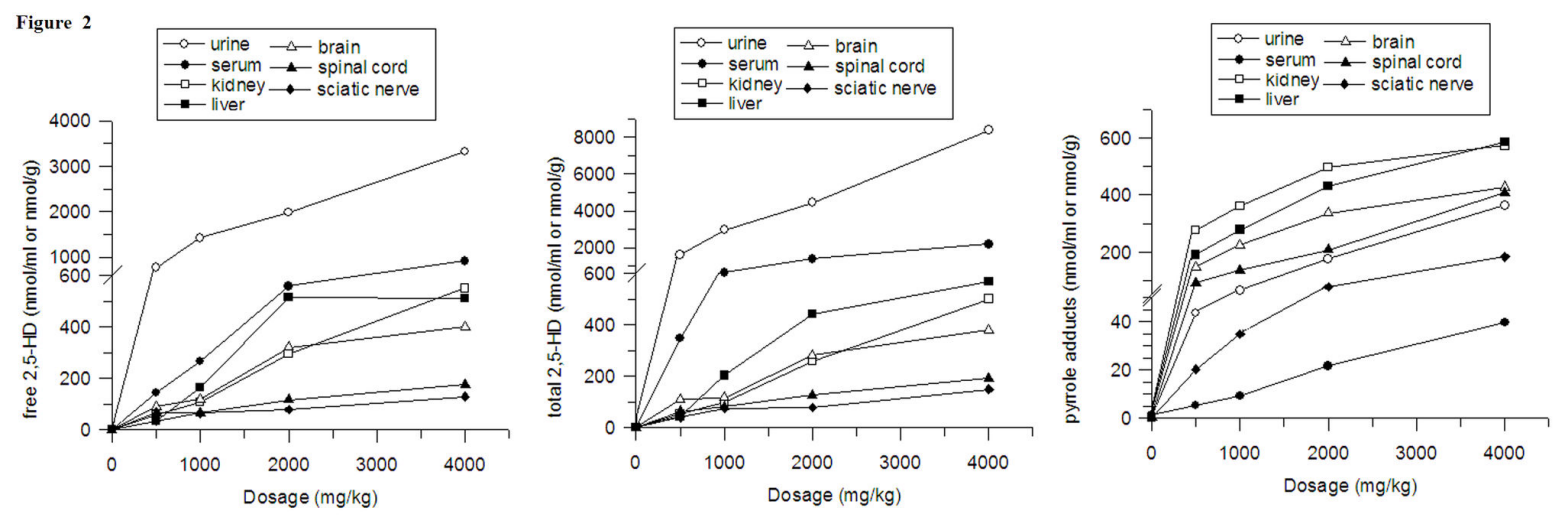
Dose-dependent effects between dosage of n-hexane and levels of free 2, 5-HD, total 2, 5-HD and pyrrole adducts in tissues (n=8).

Tissue distributions of free 2, 5-HD, total 2, 5-HD and pyrrole adducts in rats following *n*-hexane exposure, are shown in Figure 1. Similar distribution patterns were observed between free and total 2, 5-HD. Urine had the highest concentrations of free and total 2, 5-HD, followed by serum, kidney, brain, spinal cord, liver and sciatic nerve. However, pyrrole adducts showed a different distribution pattern from 2, 5-HD, with the highest concentration of pyrrole adducts observed in kidney, followed by liver, brain, spinal cord, urine, sciatic nerve and serum. Significant dose-dependent effects were observed between the dosage of *n*-hexane and the tissue levels of free, total 2,5-HD and pyrrole adducts (Figure 2), and the levels of pyrrole adducts in serum was 5.4±1.23 nmol/ml at the dose of 500 mg/kg, which was about 4 times more than that in control group (1.23 ± 0.26 nmol/ml).

### Correlations among Free 2,5-HD, Total 2,5-HD and Pyrrole Adducts within the Same Tissues

**Figure 3:**
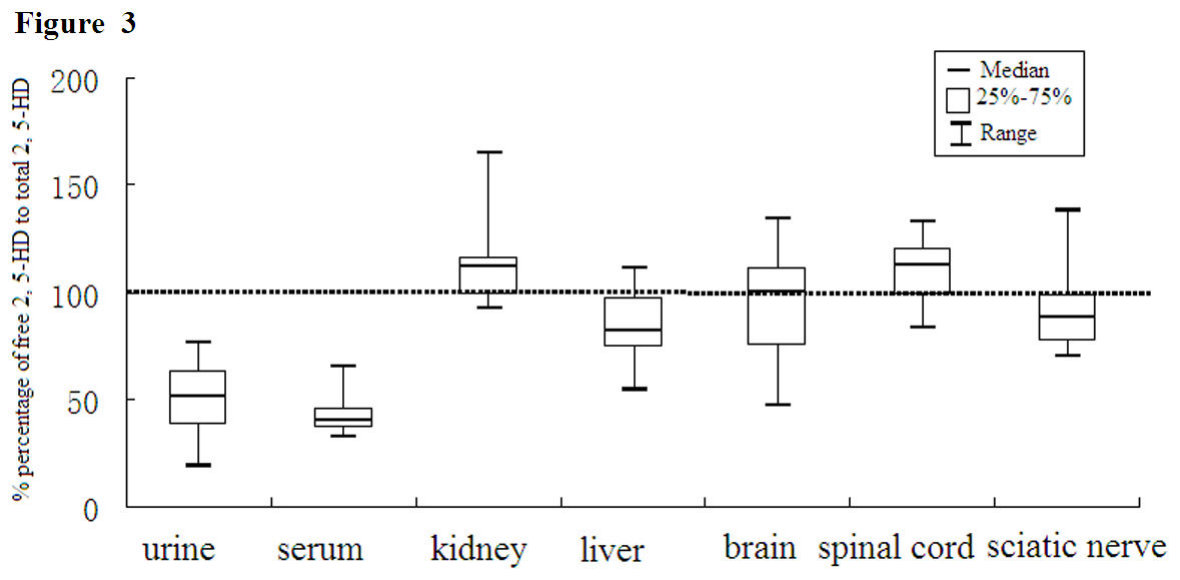
Percentage of free 2,5-HD to total 2,5-HD in different tissues of rats (n=8).

**Figure 4:**
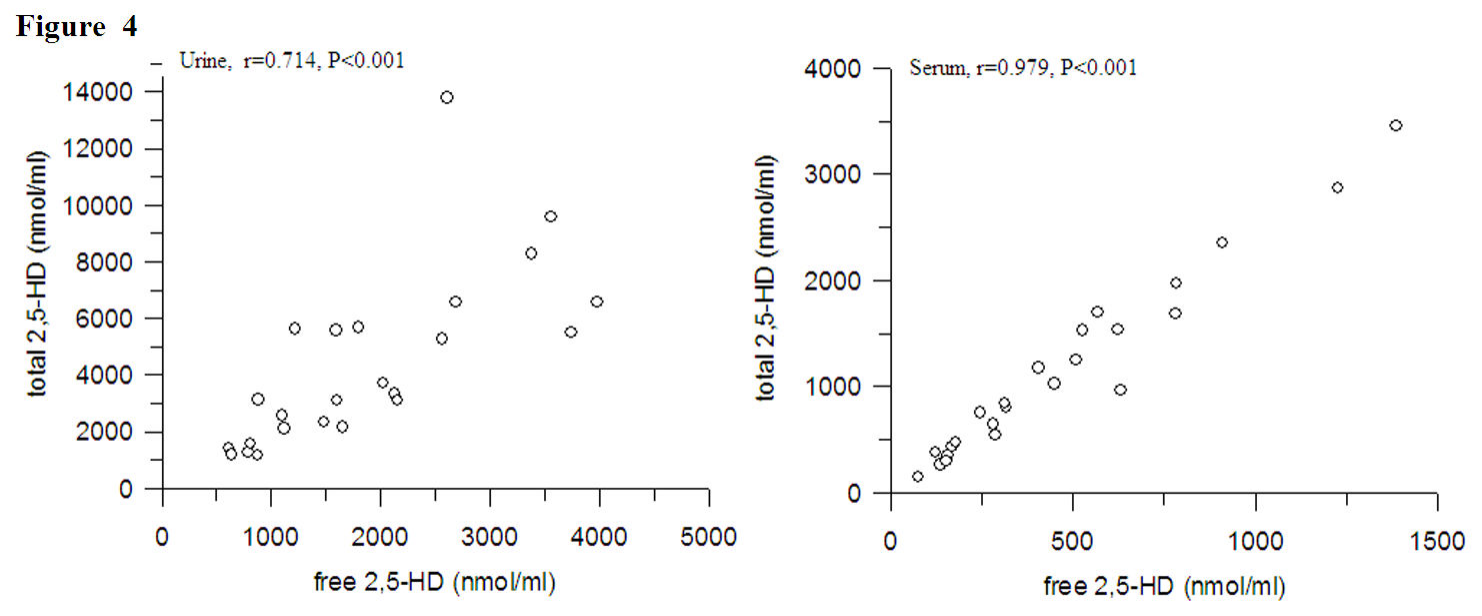
Correlations between free and total 2,5-HD in different tissues (n=8).

In order to estimated the effect of acid hydrolysis on the level of 2,5-HD in different tissues, the percentage of free 2, 5-HD to total 2, 5-HD was calculated. Significantly different percentages were observed (Figure 3). The percentage was about 50% in serum and urine, and 100% in other tissues. These results indicated that acid hydrolysis had nearly no effect on the level of 2, 5-HD in spinal cord, liver, kidney, brain and sciatic nerve. Significant correlations were observed between free 2, 5-HD and total 2, 5-HD within the urine and serum (Figure 4). The coefficient of between free 2, 5-HD and total 2, 5-HD in serum (r = 0.979, p < 0.001)was better than that in urine (r = 0.714, p < 0.001).

**Figure 5:**
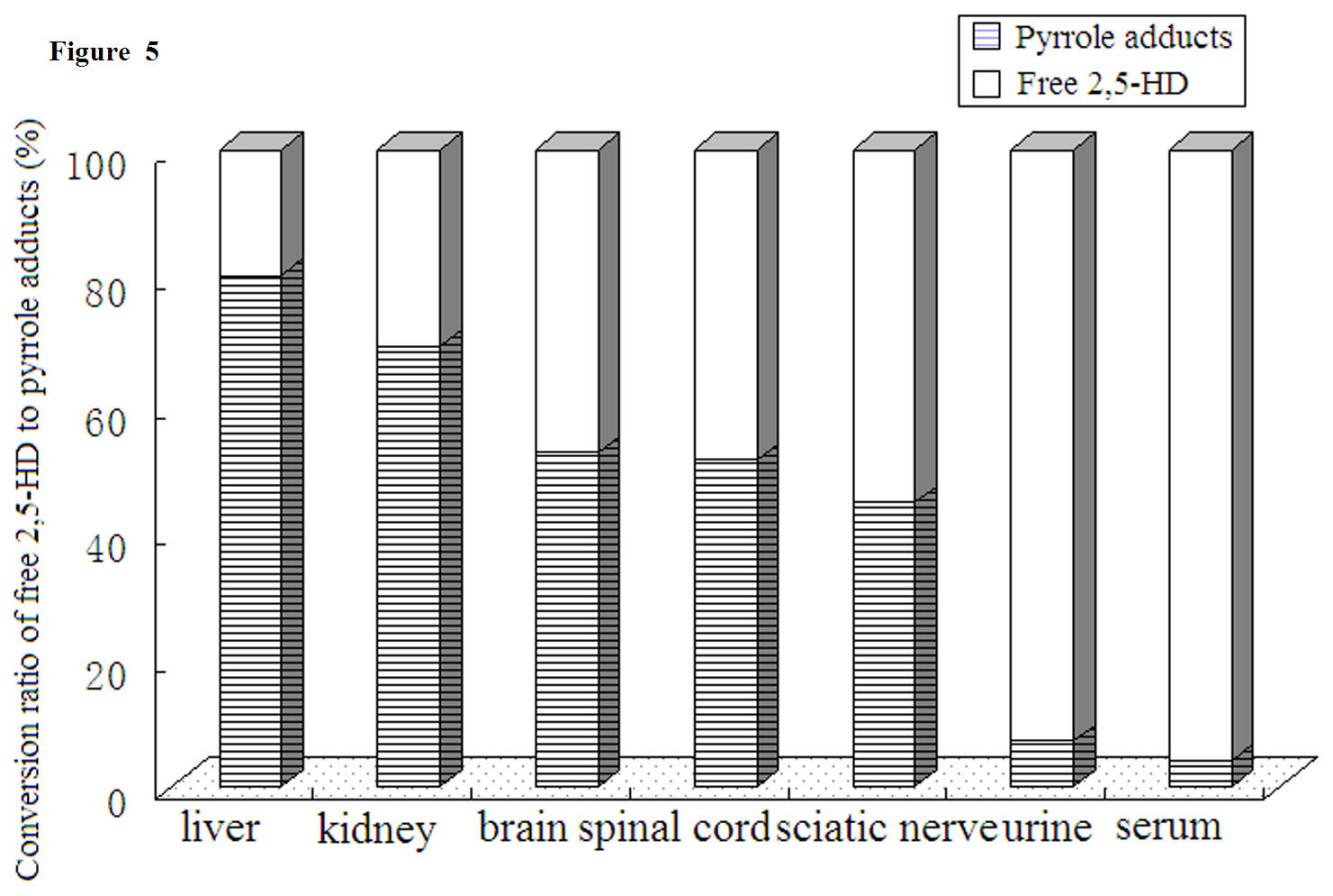
Conversion ratio of free 2,5-HD to pyrrole adducts in different tissues.

**Figure 6:**
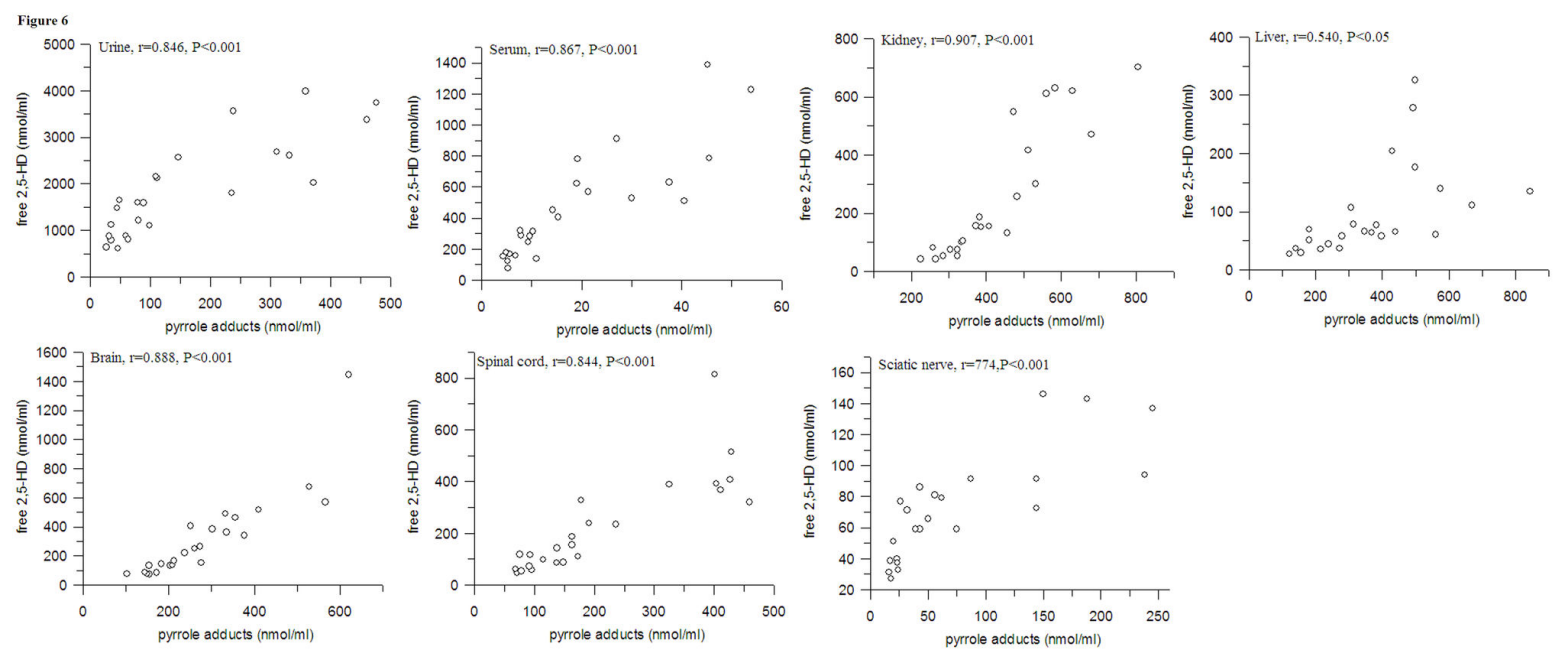
Correlations between free 2, 5-HD and pyrrole adducts in different tissues of rats (n=8).

*In vivo*, partial free 2,5-HD could be transformed to pyrrole adducts at a mole ratio of 1:1. Therefore, the conversion ratio of free 2,5-HD to pyrrole adducts, were calculated by pyrrole adducts / (free 2,5-HD + pyrrole adducts), within the same tissues. As shown in Figure 5, the highest proportion of pyrrole adducts was observed in liver, followed by sciatic nerve, kidney, spinal cord, brain, urine and serum. This result suggested that liver might be the most suitable tissues for 2, 5-HD to be transformed to pure pyrrole adducts. Significant correlations were observed between 2, 5-HD and pyrrole adducts within the same tissues (Figure 6). The most significant correlation was seen in kidney (r = 0.907), followed by brain (r = 0.888), serum (r = 0.867), spinal cord (r = 0.844), urine (r = 0.846), sciatic nerve (r = 0.774) and liver (r = 0.540), which indicated that the formation of pyrrole adducts was related to free 2, 5-HD in tissues.

### Correlations between Free 2, 5-HD and Pyrrole Adducts among Different Tissues

**Figure 7:**
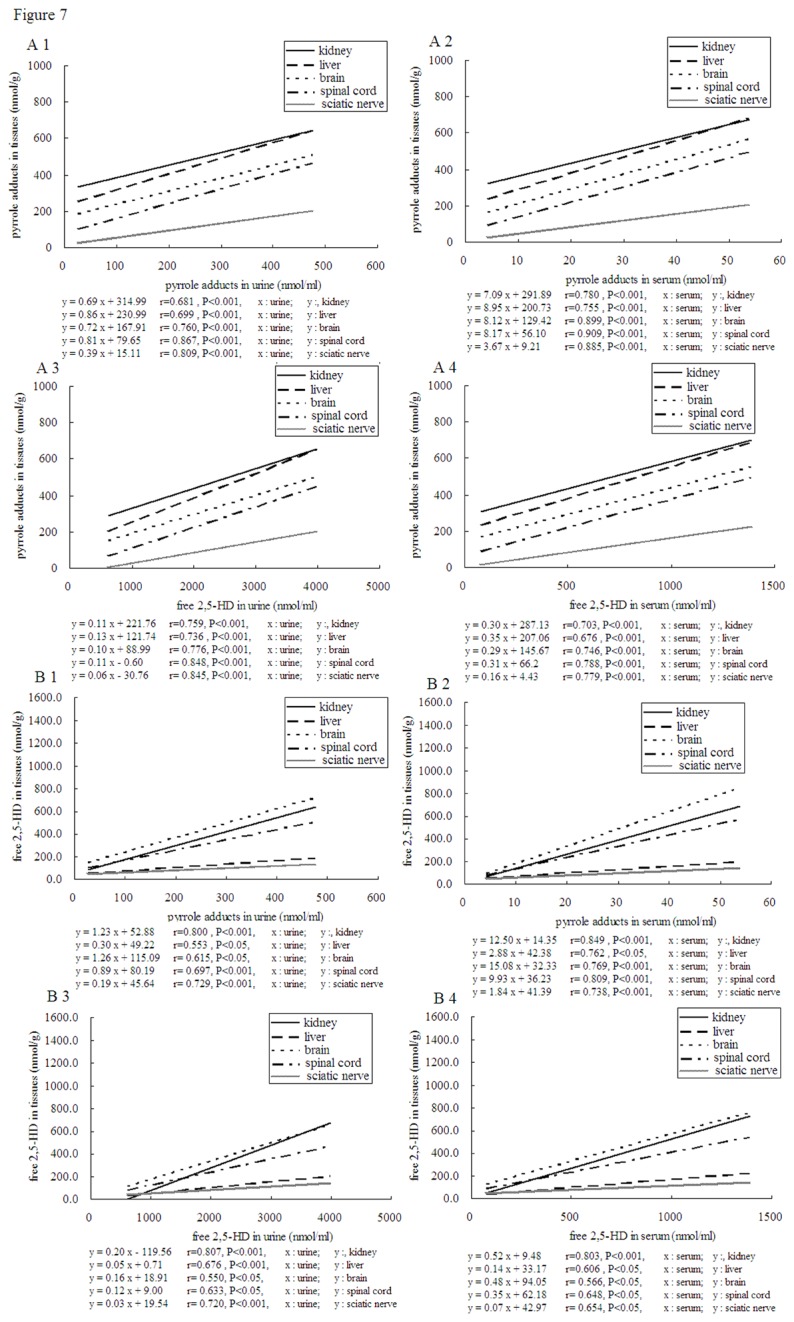
Corrections between free 2, 5-HD in urine, 2, 5-HD in serum, pyrrole adducts in urine, pyrrole adducts in seru m and free 2, 5-HD, pyrrole adducts in kidney, liver, brain, spinal cord and sciatic nerve (n=8).

Correlations between free 2,5-HD, pure pyrrole adduct in serum, urine and those in other tissues were examined by linear regression (Figure **7**). Significant correlations were found between free 2, 5-HD in urine and free 2,5-HD, pyrrole adducts in kidney, liver, brain, spinal cord and sciatic nerve. Free 2, 5-HD in serum, pyrrole adducts in urine and pyrrole adducts in serum showed similar results with free 2, 5-HD in urine, and pyrrole adducts in serum showed the most significant correlation. Among the tissues, the most significant relationship was seen between free 2, 5-HD in brain and pyrrole adducts in serum, and the least in kidney.

## Discussion

The neurotoxicity of *n*-hexane has been proven to be related with 2,5-HD, which could react with lysine ε-amino group and then form 2,5-dimethylpyrrole adducts [7,25,28-30]. The internal dose of *n*-hexane and 2, 5-HD following inhalation exposure in laboratory animals were available. *n*-Hexane could be detected in blood, liver, kidney, brain, lung, testes and sciatic nerve. The highest concentration of *n*-hexane was found in sciatic nerve and the lowest in blood. However, the distribution pattern of 2, 5-HD was different with that of *n*-hexane. The highest concentration of 2, 5-HD was seen in kidney and the lowest in liver [18,20,31].

In previous studies, it has been proven that 2,5-HD could react with many amino residues (such as amino acid, peptide and ammonia) and form many different kinds of pyrrole adducts *in vitro* and *in vivo*. These pyrrole adducts have the common features: 2,5-dimethylpyrrole ring, of which there is no other groups on the C3 and C4. These pyrrole adducts could be considered as monomeric pyrrole adducts, which could be determined by colorimetric assay with DMBA. However, these monomeric pyrrole adducts may autooxidize or react with SH groups to form polypyrrole adducts or other pyrrole derivatives. The structures of the formed pyrrole adducts after autooxidation or reaction with SH groups were still unknown. These pyrrole adducts could be considered as polypyrrole adducts. Once the polypyrrole adducts formed, the pyrrole ring may not be detectable with DMBA [13,14,16,17,32]. Thus, the pyrrole adducts referred in this study were the monomeric pyrrole adducts, not including the polypyrrole adducts or other pyrrole derivatives. The purpose of present study was to find a sensitive biomarker for *n*-hexane exposure. Although the DMBA method might underestimate the levels of pyrrole adducts, the pyrrole adducts detected by DMBA method showed a good dose-dependent effect with *n*-hexane, which suggested that this limitation could not hamper the use of pyrrole adducts detected using this method (monomeric pyrrole adducts) as a sensitive biomarker for *n*-hexane exposures. Thus, the total pyrrole adducts might be not necessary, which was similar to that free 2,5-HD could be used as a biomarker instead of total 2,5-HD (American Conference of Governmental Industrial Hygienists).

In the current study, tissue distributions of free 2,5-HD, total 2,5-HD and pyrrole adducts were determined 24 hours after the last administration. The results showed that *n*-hexane exposure increased the concentrations of free 2, 5-HD, total 2,5-HD and pyrrole adducts in a dose-dependent manner, which was consistent with previous reports [6,33]. Free 2, 5-HD and total 2, 5-HD showed similar distribution patterns, while the pyrrole adducts displayed a dissimilar pattern with them. The urine and liver contained the highest concentrations of free, total 2, 5-HD and pure pyrrole adducts, respectively, and the sciatic nerve and serum had the lowest levels of 2, 5-HD and pure pyrrole adducts, respectively. In *in vitro* studies, it had been documented that the formation of pyrrole adducts was not only related to 2, 5-HD, but also to lysine ε-amino group [7,8]. Different contents of 2,5-HD and lysine ε-amino group might lead to disparate tissue distribution patterns of free 2, 5-HD, total 2,5-HD and pure pyrrole adducts.

Free 2,5-HD was approximately 4.6-47.4% of total 2,5-HD in urine, and 50% in serum [23]. In the present study, similar results were obtained in urine and serum, while an increase of 2,5-HD by acid hydrolysis was not observed in liver, kidney, spinal cord, brain and sciatic nerves, which suggested that these tissues did not contain 4, 5-dihydroxy-2-hexanone. Thus, free 2,5-HD, not total 2,5-HD, could represent internal dose of 2,5-HD.

The formation of pyrrole adducts was considered to be related with 2,5-HD [5-10]. In the present study, the biotransformation rate of 2,5-HD to pyrrole adducts was calculated. Different rates were observed in tissues. The highest rate was observed in liver, the major metabolic site of *n*-hexane, which indicated that 2, 5-HD was more easily converted to pyrrole adducts in liver [34,35]. The reason for this might be that the level of lysine ε-amino group in liver was higher than that in other tissues. Moreover, Significant correlations were observed between 2, 5-HD and pyrrole adducts within the same tissues, which suggested that pyrrole adducts might reflect the free 2,5-HD within the same tissues [7,8].

In order to ascertain whether or not the 2,5-HD and pure pyrrole adduct in serum or urine could represent those in liver, kidney, spinal cord, brain and sciatic nerves, the correlations between free 2,5-HD, pure pyrrole adduct in serum, urine and those in these tissues were examined by linear regression. Pyrrole adducts in serum showed the most significant correlation.

In summary, tissue distribution patterns of 2,5-HD and pyrrole adducts were inconsistent. Dose-dependent effects were observed between the *n*-hexane exposure and the tissue levels of 2,5-HD, pure pyrrole adducts. Significant correlations were observed among free 2, 5-HD, total 2, 5-HD and pyrrole adducts within the same tissues. Pyrrole adducts in serum showed the most significant correlation with 2, 5-HD or pyrrole adducts in tissues, suggesting that pyrrole adducts in serum might be a better indicator for the internal doses of 2, 5-HD and pure pyrrole adducts.
